# Are lacunar infarcts associated with a “susceptibility vessel sign”? A 7-tesla magnetic resonance imaging study

**DOI:** 10.1093/esj/aakaf011

**Published:** 2026-01-01

**Authors:** Sam J Neilson, Natasha E Fullerton, Sin Yee Foo, Stephen Makin, David Porter, Keith W Muir

**Affiliations:** School of Cardiovascular & Metabolic Health, University of Glasgow, Glasgow, United Kingdom; NHS Greater Glasgow and Clyde, Department of Neuroradiology, Institute of Neurological Sciences, Glasgow, United Kingdom; NHS Greater Glasgow and Clyde, Department of Neuroradiology, Institute of Neurological Sciences, Glasgow, United Kingdom; Centre for Rural Health, University of Aberdeen, Aberdeen, United Kingdom; Department of Magnetic Resonance Imaging Physics, Centre for Cognitive Neuroimaging, University of Glasgow, Glasgow, United Kingdom; School of Cardiovascular & Metabolic Health, University of Glasgow, Glasgow, United Kingdom

**Keywords:** MRI, lacunar stroke, Small vessel disease, stroke

## Abstract

**Introduction:**

The pathophysiological basis for lacunar stroke is uncertain. The susceptibility vessel sign (SVS) on magnetic resonance imaging (MRI) is associated with thrombotic large vessel occlusion and has been reported in association with lacunar infarcts using T2* imaging. We investigated the presence of a relevant SVS in acute lacunar stroke with susceptibility-weighted imaging (SWI) and time-of-flight MR angiography (TOF-MRA) at 7 Tesla (T).

**Patients and methods:**

We performed a single-centre prospective observational study in patients with small subcortical infarct confirmed on 1.5 or 3 T MRI. Additional 7 T MRI was acquired and raters independently reviewed 7 T SWI and TOF-MRA sequences blinded to clinical data. Presence of an SVS and any associated occluded vessels were recorded. A SVS was considered present if reported by two or more raters in the relevant hemisphere with agreement confirmed at consensus review.

**Results:**

Twenty people (10 male, 10 female), with median age 67.5 [interquartile range (IQR) 64–81] years and median National Institutes of Health Stroke Scale 3 (IQR 2–4.75), underwent 7 T MRI. Possible SVS was visualized in 7 of 20 scans (35%) on SWI, with 4 considered highly likely (20%). TOF-MRA review showed an occluded small vessel proximal to the infarct in 1 of 20 patients (5%). This was not associated with a positive SVS on SWI.

**Conclusion:**

A possible SVS was observed in up to 7 of 20 (35%) people with recent small subcortical infarcts, but anatomically related vessel occlusion was not confirmed using TOF-MRA. Diagnosis of small vessel SVS appears subjective and confirmation with 3-dimensional vascular imaging may increase reliability.

## Graphical Abstract

**Graphical Abstract:**
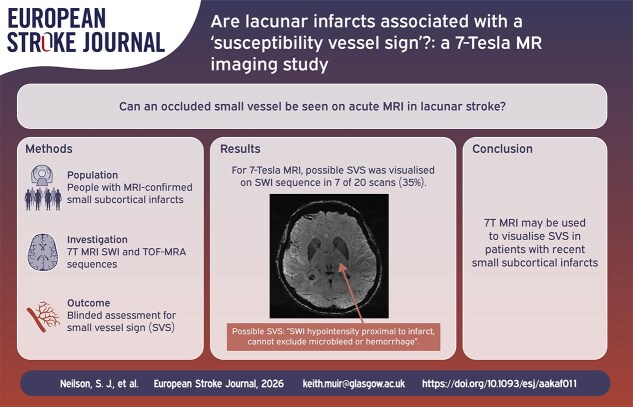

## Introduction

Lacunar stroke is a clinical syndrome most commonly related to radiologically defined recent small subcortical infarcts.^1^ Radiologically identified lacunar infarcts are a component of cerebral small vessel disease (SVD), which accounts for nearly 25% of all strokes,^2^ and is the second most common cause of dementia. The pathogenesis of lacunar infarcts is not as well understood as the pathogenesis for large vessel and cardio-embolic stroke. Clinical lacunar syndromes and the imaging finding of recent small subcortical infarcts^3^ incorporate a range of pathologies with potentially diverse underlying mechanisms. The mechanism of vessel occlusion in small vessel disease may differ from predominantly thromboembolic occlusions consequent to large vessel atheroma and cardio-embolic stroke. The differing mechanisms have potentially important implications for therapeutic intervention.^4^ Intrinsic disease of the arterioles in SVD is associated with endothelial dysfunction, vessel wall disruption and altered blood–brain barrier permeability.^5^ Medications with endothelial stabilizing properties have shown benefit in clinical trials and these would be unlikely to deliver benefit if the mechanism of occlusion was purely thrombotic.^6^ There is also limited evidence for benefit of thrombolysis compared with control in acute lacunar stroke.^7^

Some radiological lacunar infarcts may be secondary to branch atheromatous perforator obstruction^8^ while others may arise from propagation of small cardio-embolic thrombi.^9^ The mechanisms of vessel occlusion in SVD are less well established due, in part, to the small size of these perforating vessels limiting direct visualization using conventional imaging techniques. The higher resolution of 7 Tesla (T) magnetic resonance imaging (MRI) offers an opportunity to visualize smaller intracranial vessels including the lenticulostriate arteries using non-invasive time of flight MR angiography (TOF-MRA).^10^

The susceptibility vessel sign (SVS) is an MRI finding of signal loss seen on gradient-echo (GE) or susceptibility weighted imaging (SWI) corresponding to arterial thrombus causing vessel occlusion. The SVS should demonstrate a hypointense signal immediately proximal to the relevant ischaemic diffusion-weighted imaging (DWI) lesion. The SVS has been associated with cardio-embolic stroke, larger infarct and more disabling stroke;^11-13^ as with its analogue on non-contrast computed tomography, the dense vessel sign, the sensitivity of the SVS for vessel occlusion is limited since signal change depends on clot composition.^14^ The SVS has been reported in association with lacunar infarcts in retrospective studies,^15^^,^^16^ implying an underlying thrombotic occlusion in a proportion of lacunar infarcts. The development of ultra-high field MRI allows higher spatial resolution and direct visualization of relevant small vessels using both SWI and TOF-MRA.^17^ Although presence of SVS has not been extensively studied at 7 T, other research studies recording cerebral microbleeds^18^ and intracranial vessel wall imaging^19^ suggest detection of SVS may be more sensitive at 7 T than at lower field strengths.

We investigated whether obstruction of small vessels could be visualized on 7 T TOF-MRA and whether an associated SVS could be detected on SWI sequences in people with acute lacunar infarcts.

## Patients and methods

### Participants

We performed a single-centre prospective observational imaging study. We recruited adults aged 18 years and above, with capacity to provide informed consent, presenting acutely to the acute stroke service at the Queen Elizabeth University Hospital in Glasgow with symptoms of lacunar stroke. We included pure motor, or sensorimotor lacunar stroke syndromes as described by the Bamford classification^2^ accompanied by the presence of a small subcortical infarction <20 mm in axial diameter [STandards for ReportIng Vascular changes on nEuroimaging (STRIVE) criteria]^1^ within the basal ganglia or centrum semiovale on a clinical 1.5 T or 3 T MRI within 7 days of symptom onset. We excluded participants if MRI confirmed lacunar syndrome caused by brainstem infarct or if MRI demonstrated additional infarcts including separate distinct subcortical, cortical or brainstem infarct, or infarct in the contralateral hemisphere, or if participants had any established 7 T MRI contra-indications including non-fixed metallic implants, tattoos above the shoulder or cardiac pacemakers. Details of the 7 T MRI protocol are included in the appendix. Recruitment target was 20 based on funding availability. We recorded age, sex, comorbidities, blood pressure, glucose, cholesterol, medications, National Institutes of Health Stroke Scale (NIHSS)^20^ at day of recruitment and acute stroke treatment. Short-form stroke impact scale (SSIS)^21^ was recorded at day 30. The study received approval from the local research ethics committee (reference 20/WS/0012). All participants provided written informed consent prior to inclusion. This study was performed in accordance with the standards set out in the 1964 Declaration of Helsinki and its amendments.

### MRI analysis

Two experienced neuroradiologists and a stroke neurologist independently reviewed SWI and TOF-MRA blinded to details of clinical presentation. A possible SVS was deemed present if there was an area of hypointensity on the SWI sequence in the territory of an ipsilateral small vessel, immediately proximal to restricted diffusion on DWI. TOF-MRA was used to confirm the presence of small vessel occlusion. Observers graded possible SVS as either “likely SVS,” or where a cerebral microbleed or haemorrhage into the infarct could not be excluded as a cause of the susceptibility weighted artifact this was graded as “possible SVS.” Consensus review by all three raters was undertaken for all cases where there was not complete agreement. One of the neuroradiologists rated the TOF-MRA imaging quality to assess whether this may have impacted on ability to visualize lenticulostriate vessel. The quality of TOF-MRA images were reviewed by an experienced neuroradiologist and a stroke research fellow. Lenticulostriate vessels were visualized and an assessment was made as to whether movement artifact affected image quality such that potentially occluded vessel may not have been visualized, even if present. Imaging was reviewed for presence of white matter hyperintensities (WMH) which was graded using the Fazekas scale,^22^ lacunes were counted using FLAIR sequence and cerebral microbleeds (CMBs) were counted using the SWI sequence and the Microbleed Anatomical Rating Scale (MARS).^23^ In determining the likely aetiology of stroke in each case, one rater who was not blinded to MRI result recorded the ASCOD (A: atherosclerosis; S: small-vessel disease; C: cardiac pathology; O: other causes; D: dissection) phenotyping score.^24^ This assigns the degree of likelihood of stroke aetiology based on imaging and clinical features: 1 is potentially causal, 2 is causal link uncertain, 3 causal link is unlikely and 0 cause is not detected; 9 records incomplete work-up.^24^

A Mann–Whitney U test was performed to assess an association between presence of SVS and lacunes, CMBs and WMH. Fleiss’ kappa analysis was used to assess the inter-rater reliability for presence of SVS.^25^ We calculated this for definite SVS and both definite and possible SVS.

## Results

**Figure 1:**
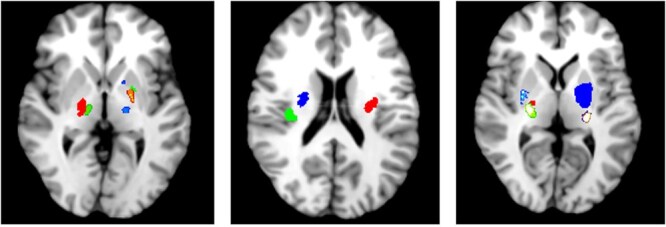
Axial magnetic resonance imaging (MRI) sample distribution of lacunar infarct location in our study population.

Between 1 August 2021 and 3 September 2022, we recruited 20 participants who underwent 7 T MRI a median of 5.5 days post-symptom onset, with a median age of 67.5 [interquartile range (IQR) 64–81] and a median NIHSS of 3 (range 0–8). Baseline imaging at 1.5 or 3 T was undertaken median 3 days post-symptoms onset and 2.5 (IQR 1–5) days prior to 7 T MRI. NIHSS was recorded at time of recruitment which was mean ± standard deviation 3.35 ± 1.4 days after symptom onset. The anatomical distribution of lacunar infarcts is demonstrated in Figure 1 with a visual representation created using Mango imaging software (http://ric.uthscsa.edu/mango/index.html).^26^ Different colours in Figure 1 represent distinct infarcts. Baseline participant characteristics, clinical and imaging outcomes are described in Table 1. Two participants received intravenous alteplase thrombolysis, two continued direct oral anticoagulants after stroke, one received 21 days of dual anti-platelet therapy and the remaining 15 received aspirin single antiplatelet at commencement of treatment.

**Table 1:** Clinical data and baseline characteristics for study participants, with clinical and imaging outcomes

Variable	*N* (%) or median (IQR)
Age (years)	67.5 (64–81)
NIHSS	3 (2–4.75)
Time between onset and 7 T MRI (days)	5.5 (2.25–8)
Left hemisphere	10 (50%)
Ischaemic heart disease	2 (10%)
Previous stroke/TIA	5 (25%)
Diabetes	6 (30%)
Peripheral vascular disease	3 (15%)
Hypertension	15 (75%)
Atrial fibrillation	2 (10%)
Smoker	6 (30%)
Clinical outcome	
SISS	23 (17.5–31)
Imaging outcomes	
Likely SVS	4 (20%)
Possible SVS	3 (15%)
Occluded vessel on TOF-MRA	1 (5%)

Abbreviations: NIHSS = National Institutes of Health Stroke Scale; TIA = transient ischaemic attack; IQR = interquartile range; SISS = short-form impact stroke scale; SVS = susceptibility vessel sign; TOF-MRA = time-of-flight-magnetic resonance imaging.

**Figure 2:**
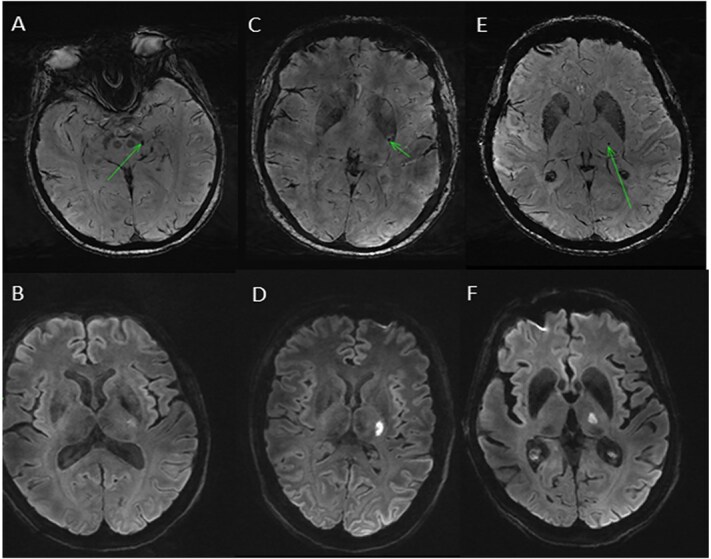
(A–F) Susceptibility-weighted imaging (SWI) demonstrating likely susceptibility vessel sign (SVS) in three different patients and associated diffusion-weighted imaging (DWI) hyperintensity representing acute infarct.

**Figure 3:**
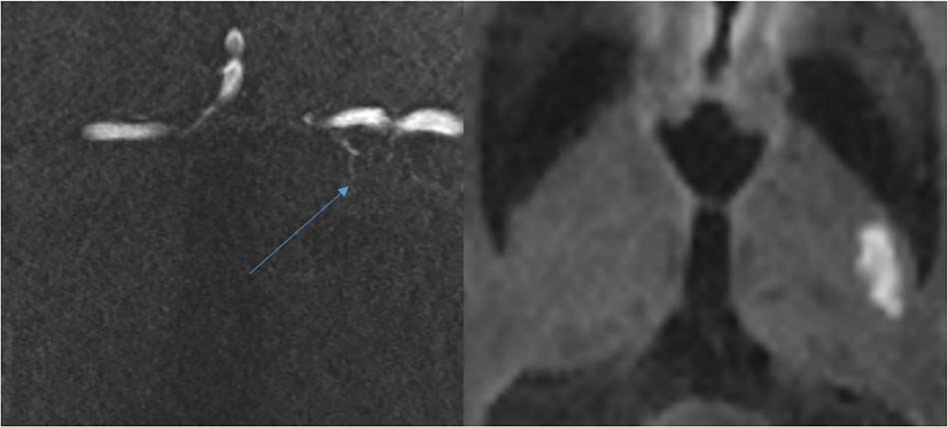
Small vessel occlusion (blue arrow) and possible associated diffusion-weighted imaging (DWI) hyperintensity on DWI sequence.

Four participants (20%) were thought likely to have a SVS on SWI (Figure 2), with 3 further study participants (15%) considered to have a possible SVS. An occluded vessel on TOF-MRA (Figure 3) could be seen in only one (5%) participant, and no associated SVS was seen on SWI in this case. The quality of TOF-MRA imaging was poor in 5 of 20 cases (25%) because of movement artifact potentially impacting on our ability to see occluded vessels. Three of the four participants with SVS seen on 7 T also had a visible SVS on prior 1.5 T or 3 T MRI. There was no significant association between presence of SVS and other SVD imaging markers (Table 2).

For the Fleiss’ kappa analysis for definite SVS among three raters the average observed agreement was 0.83, while the expected agreement by chance was 0.77. The Fleiss’ kappa coefficient κ = 0.28, suggesting fair agreement.

For the Fleiss’ kappa analysis for definite or possible SVS among three raters the average observed agreement was 0.70, while the expected agreement by chance was 0.56. The Fleiss’ kappa coefficient κ = 0.33, again suggesting fair agreement.

Of the four subjects with likely SVS on SWI, one had isolated ipsilateral carotid stenosis, one had atrial fibrillation only, and one had both carotid stenosis and atrial fibrillation. None of these cases had a visible occluded vessel on TOF-MRA. Using the ASCOD grading system (Table 3) 5 of 20 participants were found to have potentially causative (ASCOD 1) cardiac or atherothrombotic mechanisms, of which one had both. Three of these (60%) had visible SVS on SWI sequences compared with the 4 of 13 (30.8%) with visible SVS among those with only SVD as potential causal aetiology. Table 4 in the Appendix provides more detail about the individual cases, the lacunar stroke symptoms, presence of SVS, stroke aetiology, other small vessel imaging markers, and outcome measured by SSIS.

**Table 2:** Comparison of group with visible SVS on SWI and group without; all values are proportions or median (IQR)

	SVS	No visible SVS	*P*-value
*N*	7	13	
Age (years)	64 (59–85)	69 (64.5–81)	0.639
Cardioembolic (ASCOD 1)	2/7	2/13	0.643
Large vessel (ASCOD 1)	2/7	0/13	0.485
Small vessel disease (ASCOD 1)	6/7	11/13	1.0
WMH Fazekas total	2 (2–4)	4 (2–5)	0.183
Lacunes	1 (0–2)	1 (0–4)	0.699
CMBs	1 (0–4)	1 (0–2.5)	0.817

Abbreviations: SVS = susceptibility vessel sign; SWI = susceptibility-weighted imaging; IQR = interquartile range; ASCOD = (A = atherosclerosis; S = small-vessel disease; C = cardiac pathology; O = other causes; D = dissection); WMH = white matter hyperintensities; CMBs = cerebral microbleeds.

## Discussion

Our prospective study used ultra-high field MRI to image small vessels in acute lacunar stroke. We reviewed images of both SWI and TOF-MRA at 7 T. We found 4 subjects with likely SVS and 3 with possible SVS on SWI, a total of 7 of 20 (35%). None of these patients had corresponding occlusion of the perforating artery confirmed on TOF-MRA. The presence of SVS in 35% is lower than reported frequencies of SVS in large vessel occlusion which range from between 50% and 70%,^27^^,^^28^ but higher than previous studies reviewing lacunar infarct using T2*-weighted imaging at lower MR field strength.^15^^,^^16^ The presence of SVS in large vessel occlusion is indicative of clot composition with a higher proportion of red cells, which is associated with cardioembolic origin.^29^ This correlation with clot composition is likely to remain relevant in lacunar infarcts. Three of the 5 participants with potentially causal cardioembolic or large artery aetiology by ASCOD score 1 exhibited an SVS in this study compared with SVS visibility in just 4 of 13 (30.8%) with isolated SVD aetiology. This would be consistent with the aetiology of lacunar stroke being an important determinant of clot composition. Determining aetiology in lacunar infarcts is complicated by the fact that a radiological small subcortical infarct does not necessarily indicate small vessel aetiology, and the presence of SVS in these cases could be the result of thrombotic or cardioembolic small vessel occlusion from more proximal sources including the carotid and cardiac thrombus. Three of the four participants with likely SVS were found to be in atrial fibrillation or have significant carotid stenosis (>50%) or ipsilateral ulcerated plaque. In general, our study had a high proportion with co-existing potential cardioembolic or large vessel stenosis when compared with a previous study of 195 patients which found 11% with a lacunar stroke syndrome had co-existing alternative cardioembolic or large vessel stenosis aetiologies.^9^

The most likely reason for the absence of associated vessel occlusion visibility on 7 T TOF-MRA in each case where a SVS was identified is through limitations in the image quality. We found that movement artifact significantly affected the quality of images in 5 (25%) of the cases. The implicated lenticulostriate branch vessels are very small, typically <1.7 mm in diameter,^30^ making visualization difficult as has been demonstrated elsewhere.^31^ One study of 139 patients undergoing mechanical thrombectomy showing significant association between the presence of a SVS and visual inspection confirming “red-black” thrombus appearance which is associated with red blood cell–rich composition,^32^ which in turn is associated with favourable outcome.^33^ A separate study of 577 acute ischaemic stroke patients undergoing thrombectomy found the presence of a SVS to be associated with successful reperfusion^33^ and the absence of a SVS to be associated with malignancy which has been linked to a more fibrin and platelet-rich clot composition.^34^

SVS is reported much less often in association with lacunar stroke of SVD aetiology. However there are reports of detection of SVS in retrospective studies with 19% of 58 with SVS identified on a GE sequence when imaged within 24 h of stroke^16^ and 17% of a larger retrospective analysis of GE sequences in recent subcortical infarcts imaged on MRI at 1.5 T.^15^ Neither study used vascular imaging, and both reported potential challenges in differentiating SVS from cerebral microbleeds and small areas of haemorrhagic transformation into infarcts, which can have similar imaging appearances. There are studies showing that higher field strengths are more sensitive for detection of cerebral microbleeds when compared with routine clinical imaging at 1.5 T.^18^ This is also the case for visualization of enlarged perivascular spaces, small lenticulostriate vessels^35^ and cerebral microinfarcts.^36^ These findings suggest that at lower field strengths such as the 1.5 T MRI more often available in clinical practice, visualization of SVS in lacunar infarcts will be limited by spatial resolution. This will exacerbate the uncertainty resulting from the similar imaging appearances of cerebral microbleeds and small areas of haemorrhage within small infarcts.

Of the seven cases in which we were able to detect possible SVS, none had an associated occluded vessel on TOF-MRA. SWI has previously been shown to be more sensitive than TOF-MRA for visualization of large vessel occlusion, particularly for more distal middle cerebral artery occlusions,^37^ but TOF-MRA vascular imaging would be a more specific indicator of vascular thrombotic occlusion. Despite the use of ultra-high field strength MRI and TOF-MRA we were not able to confirm presence of vessel occlusion in lacunar stroke. In addition, the limitations in image quality through movement artifact, the possible explanations for the absence of occluded vessel include that clot composition in these infarcts mean they may never have been visible on TOF-MRA, or that the occluded vessel may be too small to visualize at 7 T.^31^ The 7 T imaging was performed a median 5.5 days after stroke. This timescale means that the absence of SVS on TOF-MRA may be the result of earlier complete recanalization of the initially occluded culprit small vessel. A previous imaging study noted an early imaging equivalent of SVS in lacunar stroke continued to show evidence of vessel abnormality for weeks after the initial event.^38^ A more recent study demonstrated more rapid SVS disappearance alongside, rather than before recanalization in acute embolic infarction.^39^ The statistical analysis of the between-group differences of SVS and no SVS (Table 2) are limited by small sample size.

**Table 3:** ASCOD grading for assessment of stroke aetiology^24^

ASCOD grade *n* = 20	1	2	3	0	9
Atherosclerosis (A), *n* (%)	2 (10%)	4 (20%)	8 (40%)	5 (25%)	1 (5%)
Cardiac pathology (C), *n* (%)	4 (20%)	0	0	16 (80%)	0
Small vessel disease (S), *n* (%)	17 (85%)	3 (15%)	0	0	0
Other causes (O), *n* (%)	0	0	0	0	0
Dissection (D), *n* (%)	0	0	0	0	0

The ability to detect the SVS in lacunar stroke may offer insights into the pathophysiology of SVD, an aetiological category that is based on clinical syndromes, and imaging features such as anatomical location and size, but may cover heterogeneous mechanisms. Improved mechanistic understanding in turn may allow interpretation of therapeutic strategies, both for acute treatment (response to thrombolytic drugs being uncertain) and secondary prevention (combination antiplatelet therapy being higher risk compared with other aetiologies). Our data suggest that a high proportion of patients may exhibit the SVS when imaged at high field strength. The presence of the SVS in lacunar stroke is associated with underlying embolic sources, including cardio-embolic or from carotid atheromatous disease, while showing no firm associations with other imaging markers of SVD.

## Supplementary Material

aakaf011_Supplementary_materials
