# Supplementary material for: Are lacunar infarcts associated with a “susceptibility vessel sign”? A 7-tesla magnetic resonance imaging study
Source: Eur Stroke J. 2026 Jan 1;11(1):aakaf011. doi: 10.1093/esj/aakaf011 (PMC12866648; doi:10.1093/esj/aakaf011)
Supplement: aakaf011_Supplementary_materials [file aakaf011_supplementary_materials.docx]

Supplementary materials.

MRI protocol

7 tesla MRI was acquired on a Siemens Magnetom Terra (Siemens Healthineers, Erlangen, Germany) using a commercial Nova Medical Head Coil 1TX / 32RX (Nova Medical, Wilmington, MA). Sequences included 3-dimensional T1-weighted imaging (mp2rage) (40), T2 FLAIR (fluid-attenuated inversion recovery), SWI, Diffusion weighted imaging (DWI) (Resolve)(41), and TOF-MRA with a slab centred on lenticulostriate vessels.

Table 4. Representation of stroke syndromes, presence of SVS, potential aetiologies (ASCOD), imaging evidence of small vessel disease markers and 30 day Short form Stroke Impact scale (SSIS).

| **ID** | **Lacunar stroke symptoms** | **Age** | **NIHSS** | **Consensus SVS on SWI** | **A** | **S** | **C** | **O** | **D** | **Fazekas total** | **Lacune**  **count** | **CMBs** | **Day 30 SSIS** |
| --- | --- | --- | --- | --- | --- | --- | --- | --- | --- | --- | --- | --- | --- |
| 1 | Motor and Sensory | 60 | 2 | Possible SVS | 3 | 1 | 0 | 0 | 0 | 4 | 1 | 0 | 21 |
| 2 | Motor | 64 | 2 | Possible SVS | 3 | 1 | 0 | 0 | 0 | 2 | 0 | 1 | 32 |
| 3 | Motor | 80 | 2 | Probable SVS | 1 | 2 | 1 | 0 | 0 | 2 | 0 | 0 |  |
| 4 | Motor | 64 | 2 |  | 3 | 1 | 0 | 0 | 0 | 3 | 0 | 0 | 14 |
| 5 | Motor | 81 | 5 |  | 2 | 1 | 0 | 0 | 0 | 5 | 5 | 3 | 16 |
| 6 | Motor | 85 | 5 | Possible SVS | 1 | 1 | 0 | 0 | 0 | 3 | 2 | 0 | 13 |
| 7 | Motor | 59 | 4 | Probable SVS | 2 | 1 | 0 | 0 | 0 | 2 | 2 | 1 | 19 |
| 8 | Motor and Sensory | 69 | 0 |  | 2 | 1 | 0 | 0 | 0 | 5 | 1 | 2 | 35 |
| 9 | Motor and Sensory | 59 | 8 |  | 9 | 1 | 0 | 0 | 0 | 2 | 0 | 2 | 22 |
| 10 | Motor | 65 | 2 |  | 2 | 1 | 0 | 0 | 0 | 4 | 1 | 1 | 34 |
| 11 | Motor and Sensory | 53 | 6 | Probable SVS | 0 | 1 | 0 | 0 | 0 | 1 | 0 | 4 | 23 |
| 12 | Motor and Sensory | 83 | 4 |  | 0 | 1 | 1 | 0 | 0 | 4 | 0 | 1 | 13 |
| 13 | Sensory | 65 | 1 |  | 0 | 2 | 0 | 0 | 0 | 2 | 0 | 0 | 35 |
| 14 | Motor and Sensory | 75 | 4 |  | 3 | 1 | 0 | 0 | 0 | 5 | 3 | 19 | 23 |
| 15 | Motor and Sensory | 69 | 4 |  | 3 | 2 | 0 | 0 | 0 | 2 | 0 | 0 | 30 |
| 16 | Motor | 81 | 3 |  | 0 | 1 | 0 | 0 | 0 | 6 | 7 | 20 | 20 |
| 17 | Motor and Sensory | 66 | 6 |  | 0 | 1 | 0 | 0 | 0 | 3 | 5 | 0 |  |
| 18 | Motor | 64 | 3 |  | 3 | 1 | 1 | 0 | 0 | 5 | 1 | 1 | 25 |
| 19 | Motor | 91 | 3 |  | 3 | 1 | 0 | 0 | 0 | 2 | 2 | 0 | 26 |
| 20 | Motor and Sensory | 95 | 2 | Probable SVS | 3 | 1 | 1 | 0 | 0 | 5 | 3 | 5 |  |
